# Prefrontal brain function in patients with chronic insomnia disorder: A pilot functional near-infrared spectroscopy study

**DOI:** 10.3389/fneur.2022.985988

**Published:** 2022-12-15

**Authors:** Haiyan Gong, Hui Sun, Yeyang Ma, Yaling Tan, Minglong Cui, Ming Luo, Yuhui Chen

**Affiliations:** ^1^Department of Internal Neurology, Tongji Hospital, Tongji University, Shanghai, China; ^2^Department of Geriatrics, Tongji Hospital, School of Medicine, Tongji University, Shanghai, China

**Keywords:** chronic insomnia disorder, functional near-infrared spectroscopy, prefrontal cortex, Pittsburgh Sleep Quality Index, verbal fluency test

## Abstract

**Purpose:**

Insomnia is one of the most common diseases in elderly patients, which seriously affect the quality of life and psychological state of patients. The purpose of this study was to investigate the changes in the functional network pattern of the prefrontal cortex in patients with chronic insomnia disorder (CID) after taking drugs, using non-invasive and low-cost functional neuroimaging with multi-channel near-infrared spectroscopy (fNIRS).

**Methods:**

All subjects were assessed using the Pittsburgh Sleep Quality Index (PSQI), Hamilton Depression Scale (HAMD), Hamilton Anxiety Scale (HAMA), and fNIRS. The fNIRS assessment consists of two parts: the verbal fluency test (VFT) task state and the resting state, which assessed the differences in prefrontal activation and functional connectivity, respectively.

**Results:**

A total of 30 patients with chronic insomnia disorder (CID) and 15 healthy peers completed the study. During the VFT task, a significantly lower PFC activation was observed in patients with insomnia compared to the control group (*P* < 0.05). However, the PFC activation in patients taking medication was higher than in patients who did not receive medication. Functional connectivity analysis showed a weaker mean PFC channel connectivity strength in patients with CID who did not receive drug treatment. Drug treatment resulted in enhanced functional connectivity of the prefrontal lobe, especially the DLPFC and frontal poles.

**Conclusion:**

A weak prefrontal cortex response was detected in patients with CID when performing the VFT task, which could be enhanced by taking hypnotics. The weakened right prefrontal lobe network may play a role in the development of CID. fNIRS may serve as a potential tool to assess sleep status and guide drug therapy.

## Introduction

Insomnia is one of the most common sleep disorders. Epidemiological studies show that 45.4% of the respondents in China experienced varying degrees of insomnia in the past month (1). This phenomenon is more widespread in the elderly population. The overall incidence of insomnia among the elderly in China is 47.2%, while the incidence of insomnia among the elderly in the community ranges from 35 to 65% (2). Insomnia has a severe impact on patients' moods and quality of life, resulting in depression, anxiety, and other negative emotional states (3). Drug therapy is the current mainstay of insomnia treatment, including sedative-hypnotic benzodiazepines and non-benzodiazepines, melatonin receptor agonists, sedative antidepressants, sedative antihistamines, and orexin receptor antagonists (DORAs). The choice of drug is often based on an assessment of the patient's clinical symptoms, such as difficulty falling asleep, waking up in the middle of the night and staying awake, or waking up early. However, this approach is not always effective, as patients with similar symptoms exhibit varied responses to drug treatment. Symptom-based drug selection to relieve insomnia symptoms is not an objective method. In addition to drug treatment, neuromodulation may also regulate sleep structure and improve sleep quality. Neuromodulation techniques mainly include transcranial magnetic stimulation (TMS), transcranial electrical stimulation (TES), and acupuncture. These new approaches improve sleep quality by altering brain excitability but require a comprehensive assessment of brain function.

Insomnia disorder is related to cerebral cortical dysfunction (4). Recent evidence indicates that the left dorsal and medial frontal regions may be particularly important in regulating sleep (5). Electroencephalography (EEG), fNIRS, and functional magnetic resonance (fMRI) studies have demonstrated that the prefrontal lobe plays a crucial role in maintaining sleep (6–8). Furthermore, EEG-related studies have demonstrated the relationship between prefrontal neural activity and sleep. Joy Perrier et al. studied the prefrontal neural activity in patients with primary insomnia, who exhibited a higher beta power spectrum and lower delta power spectrum (9). The fNIRS study showed that prefrontal excitability varied across sleep stages in normal subjects. The oxyhemoglobin (HbO) and total hemoglobin (HbT) levels in the rapid eye movement (REM) stage were lower than those in other sleep stages and waking stages. The neural activity of the prefrontal cortex is related to sleep regulation and maintenance (10). Cortical and nuclear effects can be studied with high precision due to the high spatial resolution of fMRI. In addition, hyperresponsiveness to stimuli has been observed in the precentral cortex, prefrontal cortex, and default mode network (11). Numerous neuroimaging studies have demonstrated that changes in the excitability of the prefrontal cortex and its association with other cortex (nuclei) play key roles in sleep control, and alterations in these states may induce sleep disturbances (12). Sleep results from the coordinated regulation of multiple brain regions, including the frontal, parietal, and occipital lobes. The arousal state is jointly controlled by the default network, cognitive control network, salience network, and negative emotion network formed by these cortical interconnections (9). The right insula, left inferior frontal gyrus triangle, left frontal pole, right upper parietal, right medial orbitofrontal cortex, and right supramarginal gyrus form various networks, which have their own functions and local networks during sleep. They also play an essential role in sleep control (10).

Functional neuroimaging with multi-channel near-infrared spectroscopy is a potential clinical prefrontal function assessment tool, providing convenient and quick evaluation compared with previous imaging tools for insomnia research. Using fNIRS to assess prefrontal lobe function in patients with insomnia may be a potential means to investigate the mechanism of insomnia and guide clinical treatment. Increased excitability of the left prefrontal cortex contributes to sleep onset and sleep maintenance. We used fNIRS to assess whether patients had increased excitability in the left prefrontal cortex after taking the drug. In addition, we hypothesize that the drug improves the connections between different brain regions in the prefrontal cortex to help sleep.

## Materials and methods

### 2.1 Participants

Patients with CID were recruited from the neurology clinics and wards of Tongji University Affiliated Hospital. The patients were diagnosed according to the criteria of “Guidelines for the Diagnosis and Treatment of Insomnia in Adults in China (2017 Edition)” (1). The patients had a good cognitive function, had no impairments in daily living activities, and cooperated to complete all tests. Exclusion criteria included a Mini-mental State Examination (MMSE) score of < 27 points (11), anxiety or depression-related disorders diagnosed by a professional doctor, serious physical and mental illnesses that prevented the evaluation, alcoholism, or intake of neuropsychiatric drugs within 3 months, and brain lesions found in MRI. This study was approved by the Ethics Committee of the Tongji University Affiliated Hospital (approval K-2020-026). All subjects understood the study and signed informed consent.

### 2.2 Protocol and devices

**Figure 1:**
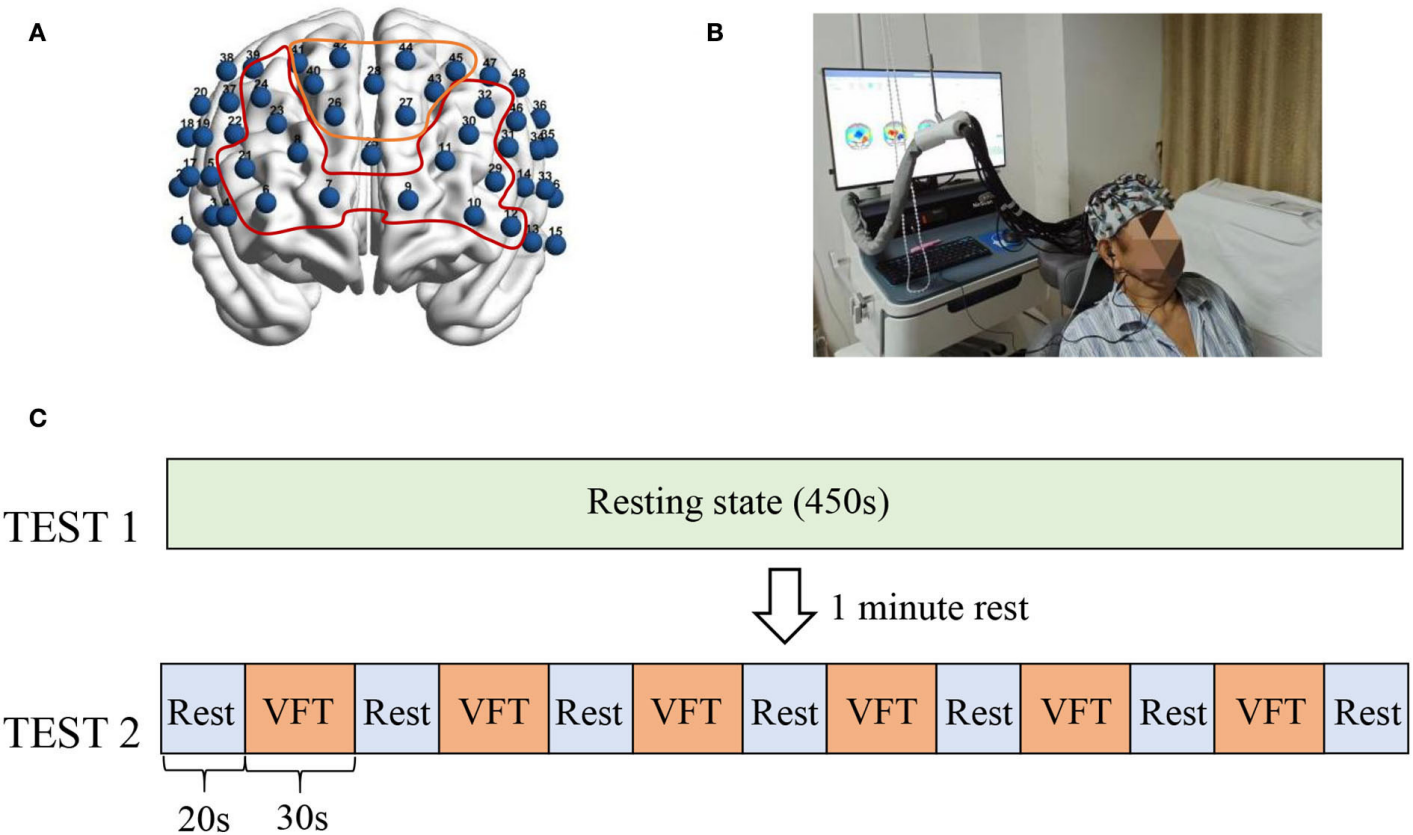
fNIRS channel location and experimental paradigm. **(A)** Shows the brain regions corresponding to the 48 channels, with the numbers indicating the channel numbers. The yellow curve corresponds to the frontopolar area and its channels (CH41, CH40, C42, CH26, CH28, CH44, CH27, CH43, and CH45). The red area indicates the dorsolateral prefrontal cortex and its covered channels (CH6, CH7, CH21, CH8, CH23, CH24, CH9, CH10, CH11, CH29, CH30, CH32, and CH12). **(B)** Shows a subject being tested by fNIRS. **(C)** Shows the test paradigm. The fNIRS assessment was divided into two parts. The 450's resting state (TEST1) was performed first, and the VFT task state was performed after a 1-min rest. The task state included a total of six blocks, each consisting of 30's to execute the VFT task and 20's to rest.

The functional neuroimaging with multi-channel near-infrared spectroscopy device used in this study was the NirScan6000B (Danyang Huichuang Medical Equipment Co., Ltd., Jiangsu, China). The fNIRS head cap includes 24 emitter optodes and 20 receiver optodes, providing a total of 48 channels. The magnetic source navigation device Nirspace (Danyang Huichuang Medical Equipment Co., Ltd., Jiangsu, China) was used to determine the corresponding brain region for each channel to localize the optode on a standard brain (Figure 1A).

To reduce the experimental error, the subjects were asked to lie on the rehabilitation treatment bed comfortably with their face up. The participants were requested to minimize body movements such as facial movements and frequent eye blinks and try to avoid any noise during data recording. In addition, the researchers observed the head, torso, and other movements (Figure 1B). After wearing the fNIRS device, the participants were subjected to a 450-s resting-state assessment, followed by a verbal fluency test (VFT) task-state assessment. The VFT task-state assessment required subjects to list words within the range of the cue within 30 s according to the voice prompt, followed by a 20-s rest. This step was repeated six times (Figure 1C). After the fNIRS assessment, professional rehabilitation physicians evaluated the patients with scales, including the Pittsburgh Sleep Quality Index (PSQI), Hamilton Depression Scale (HAMD), and Hamilton Anxiety Scale (HAMA) (12–14).

### 2.3 Data analysis and statistics

This study adopted a per-protocol set (PPS), and patients who successfully completed all assessments were included in the final data analysis. The basic conditions of the three groups before treatment were analyzed. Binary data such as gender were analyzed using the exact probability method. The W-test method and the F-test were used to confirm that the age, PQSI, HAMA, and HAMD followed a normal distribution and to verify the homogeneity of variance. The differences among the three groups were compared using an analysis of variance. If there was a statistical difference, a *t*-test was used to compare between the groups.

First, quality control of the fNIRS data was performed using FC-NIRS2.1 (https://www.nitrc.org/projects/fcnirs/), and subjects or channels with large artifacts were removed (15). For resting-state data, the preprocessing function in FC-NIRS was used, with band-pass filters set to 0.1–0.08 Hz and using PCA-OD for motion detection. Pearson's correlation analysis was performed on 400 s of data from the middle section, and the Z-value obtained was tested for the hypothesis. For task-state data, activation analysis was performed using the MATLAB-based toolkit NIRS-SPM (https://www.nitrc.org/projects/nirs_spm/). Gaussian smoothing with a full width at half maximum (FWHM) of 4 s was used as a pre-coloring parameter to correct the short-time series correlation. Furthermore, a discrete cosine transform (DCT) with a period length of 128 s was used as the cutoff frequency parameter to remove the long-term trend of the sequence. The above parameters were used as the general linear model (GLM) with the design matrix established according to the experimental conditions. A mapping algorithm was used to estimate the model (estimation). Based on the above beta and residual error estimations, a one-tailed *t*-test was used for statistical inference. The change in hemodynamic oxyhemoglobin (ΔOxyHb) was determined, and *P* < 0.05 was considered statistically significant (16). Task-state data were corrected for multiple comparisons using the tube formula multiple comparison correction in NIRS_SPM.

## Results

A total of 45 subjects participated in the study and completed all assessments. Table 1 shows the demographic characteristics of patients with and without medication for CID. The age range was 60 to 84 years, including 16 (53.3%) female subjects and 14 (47.7%) male subjects. The subjects had been suffering from insomnia for a long duration (mean 23.8 months). Among them, 15 subjects were taking sedatives and hypnotic drugs, and all felt that the drugs alleviated their insomnia. No significant differences in demographic and clinical characteristics were found between the two groups.

**Table 1:** CID group and treatment CID group demographics.

**Variable**	**HC group**	**CID group**	**Treatment CID group**	***P***
Age (years)	69.8 ± 7.7	71.1 ± 6.0	70.4 ± 6.4	0.71
Sex (F/M)	7/8	9/6	7/8	0.46
Insomnia duration	/	25.4 ± 23.2	22.1 ± 18.6	/
PSQI	4.2 ± 3.1	16.1 ± 2.4	13.4 ± 3.5	0.02^*^
HAMA	2.0 ± 2.0	8.9 ± 4.8	7.2 ± 3.2	0.01
HAMD	2.0 ± 2.0	8.2 ± 4.7	6.4 ± 3.1	0.02

* *P* < 0.05.

The PSQI of patients with CID was significantly higher than that of healthy controls. Moreover, the PSQI of patients who were on drug treatment was lower than that of the patients who were not, indicating that drugs can improve the symptoms of insomnia. Similarly, higher HAMA and HAMD scores were found in patients with CID compared to healthy subjects. However, whether patients with CID took medication or not had little effect on the results. There was no statistical difference between the two groups.

**Figure 2:**
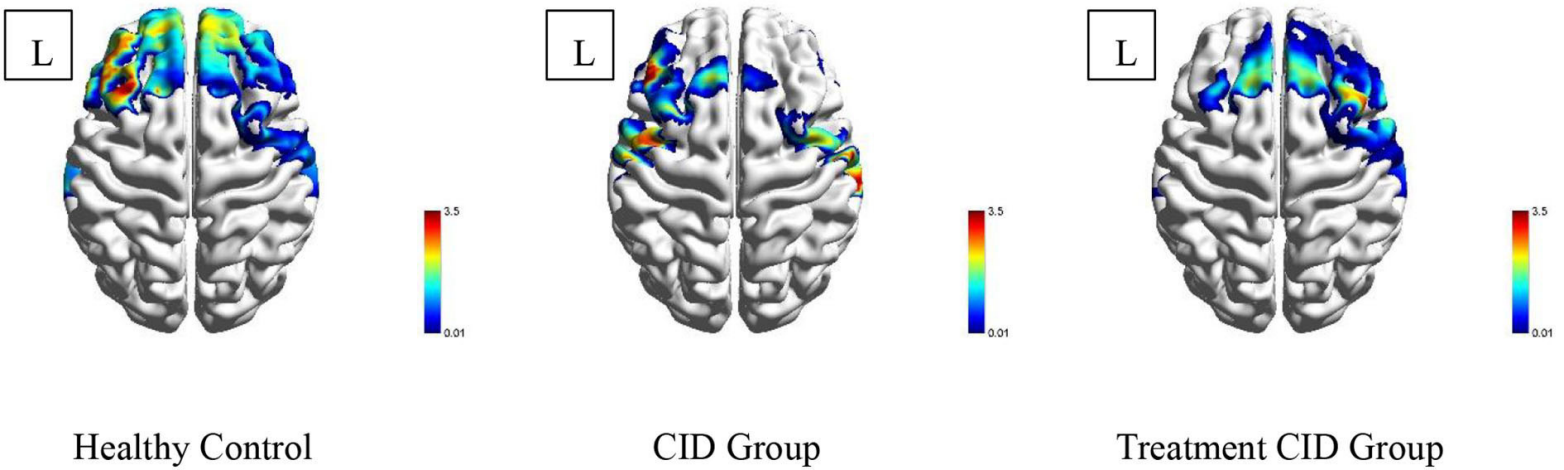
Activation of the prefrontal lobe of the three subject groups. Different colors in the figure represent the corresponding *T*-values.

When performing the VFT task, significant differences in prefrontal cortex activation were observed among the three subject groups. During the VFT task, healthy subjects exhibited extensive activation of the prefrontal cortex, particularly in the dorsolateral and frontopolar regions. In contrast, the activation of the prefrontal lobe in patients with CID without drug treatment was significantly reduced compared with the normal subjects, with no activation in the frontal pole and the dorsolateral side of the right prefrontal lobe. In patients with CID on drug treatment, lower activation of the prefrontal cortex was observed compared with the normal subjects, but the frontal pole and the right dorsolateral prefrontal cortex were significantly activated (Figure 2).

**Figure 3:**
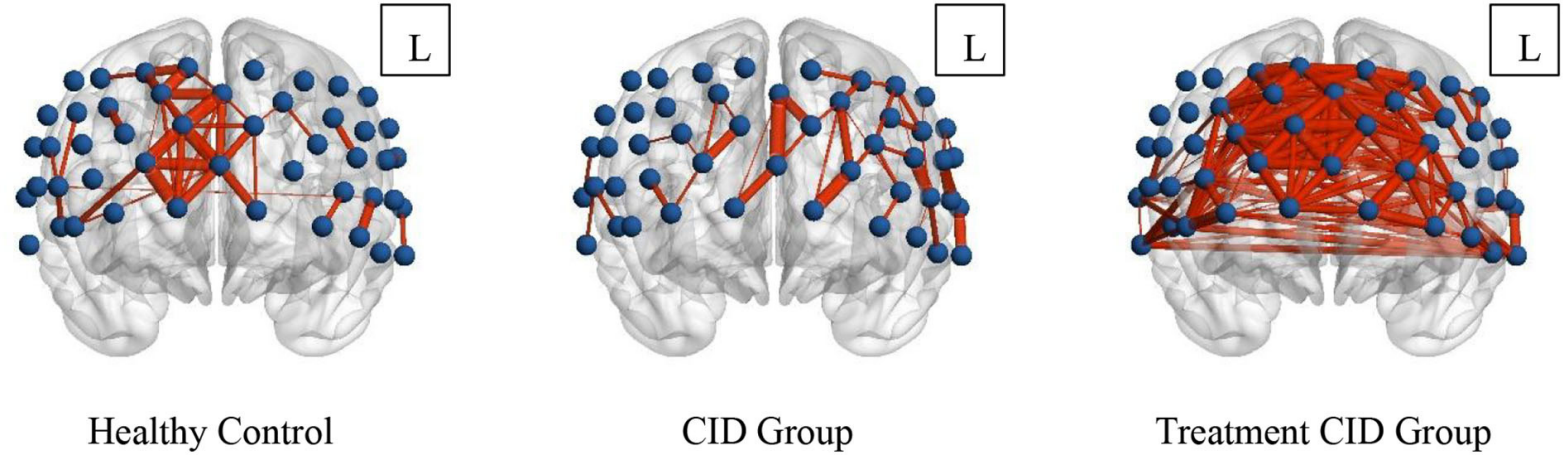
Prefrontal functional connectivity of subjects in the three groups. The blue balls in the figure represent the channels, and the red lines represent the correlation between the channels. The width of the line is proportional to the correlation coefficient.

When the sparsity threshold was set to 0.8, the mean channel connectivity strength of the prefrontal cortex was weaker in patients with insomnia and no medication, which was significantly different from normal subjects. In normal subjects, the right frontal pole and dorsolateral prefrontal cortex showed high local channel connection strength. However, patients taking drug treatment showed not only a significantly higher internal functional connectivity in the right frontal pole and dorsolateral prefrontal cortex but also the average channel connection in the entire prefrontal cortex (Figure 3).

Spearman's correlation analysis was used to statistically analyze the PSQI and the number of prefrontal cortex activation channels in patients with insomnia. The correlation coefficient was 0.322, and the Spearman rank correlation was not significant, which could not explain the correlation between the number of prefrontal cortex channel activation and the degree of insomnia. The significance level is 0.2 > 0.05, indicating that the relationship between the two variables is not significant.

## Discussion

This study demonstrated that prefrontal activation was attenuated in patients with CID compared with healthy subjects, which was consistent with previous findings (17). Jing-Jing Sun et al. showed that patients with CID exhibited low PFC activation when performing cognitive tasks. Moreover, patients with insomnia taking medication showed improved prefrontal activation but still did not reach the level of normal subjects. In addition, the mean channel functional connectivity of the prefrontal lobe was significantly decreased in patients with insomnia. However, the patients treated with hypnotics showed significantly enhanced mean channel functional connectivity, which was even higher than that of normal subjects. Therefore, one of the targets of drug therapy likely promotes sleep. It is worth noting that the results of the study showed that the improvement in sleep quality was not related to the size of the prefrontal cortex activation area. Normal cortical function plays a crucial role in sleep, and global or local dysfunction may lead to insomnia. Several brain networks, such as the default network, salience network, cognitive control network, and negative emotion network, are closely related to insomnia. The prefrontal lobe is an essential structure of the brain. It collaborates with other brain regions to maintain the human brain in the resting state and takes part in the integration of internal and external environmental information, emotional integration, and episodic memory retrieval. In clinical studies utilizing high-frequency TMS and anodal tDCS, increased PFC excitability was shown to enhance sleep therapy (18–20). Ellemarije Altena et al. used fMRI to study the prefrontal function in patients with CID, while performing VFT tasks, revealing low activation of the medial and inferior prefrontal cortical areas (Brodmann Area 9, 44, 45), which objectively demonstrated the importance of PFC in sleep (21). Perrier et al. used EEG to study the differences in prefrontal cortex functional connectivity between patients with CID and normal subjects. The β1 power spectrum of the prefrontal cortex was lower in patients with primary insomnia compared with normal people, which is also consistent with the results of this study (22). The normalization of the prefrontal cortex function has important significance in guiding the treatment of CID and evaluating the function of the prefrontal cortex may be a potential method to evaluate the efficacy of sleep therapy.

Notably, the functional network complexity of patients with drug-treated stroke was greater than that of healthy subjects, which may be a form of frontal hyperconnectivity. Hyperconnectivity refers to the paradoxical increase in functional connectivity between network regions resulting from damage to neural systems (23). It is generally considered to be a compensatory mechanism for brain dysfunction. Compared with the conventional functional network, the hyperconnectivity network is decentralized, which may be attributed to greater resource requirements to achieve normal functions. In this study, the functional network connections of the prefrontal lobe in patients with CID were reduced, and the brain network complexity was significantly increased after drug treatment. This may be due to the drug mobilizing more prefrontal lobe resources to complete sleep. However, this hypothesis is only an assumption, and complex network analysis methods are needed to verify this conjecture.

In this study, fNIRS showed good sensitivity and specificity in assessing the function of the prefrontal cortex in patients with insomnia, which is consistent with previous studies. fNIRS has certain advantages in the evaluation of cortical function and can be used as a routine tool for the evaluation of insomnia. fNIRS is more cost-effective than other commonly used neuroimaging assessment techniques, such as fMRI and EEG. The low testing cost, simple site requirements, and simple operation allow fNIRS to be used in multiple clinical situations. More importantly, the fNIRS evaluation is quick, and patients can be repeatedly evaluated during the treatment process to identify patterns, providing objective biological markers for treatment. The application of fNIRS in CID assessment is still in the preliminary exploratory stage. To verify its potential, whole-brain fNIRS should be performed to comprehensively assess various network functions involved in CID, and the results should be compared with previous studies. Identifying specific biomarkers will help develop the role of fNIRS in the evaluation and treatment of CID.

## Data availability statement

The raw data supporting the conclusions of this article will be made available by the authors, without undue reservation.

## Ethics statement

The studies involving human participants were reviewed and approved by Ethics Committee of Tongji Hospital Affiliated to Tongji University. The patients/participants provided their written informed consent to participate in this study.

## Author contributions

YC and ML contributed to the study design. HG and HS collected the baseline data, performed the statistical analysis, and interpretation of the results. YM, YT, and MC were responsible for recruiting subjects. All authors contributed to the article and approved the submitted version.
